# Online communities as arenas of “amateur expertise”: examples from the social media activity for justice for Roman Zadorov

**DOI:** 10.3389/fsoc.2024.1455130

**Published:** 2024-12-03

**Authors:** Azi Lev-On

**Affiliations:** School of Communication, Ariel University, Ariel, Israel

**Keywords:** online community, discourse, activism, experts, social media

## Abstract

**Introduction:**

This study examines online communities as arenas where diverse forms of expertise converge to influence discourse and public opinion. Using the case of social media activism advocating for justice in the wrongful conviction of Roman Zadorov for the murder of Tair Rada, it highlights how these communities serve as platforms for “professional amateurs” and demonstrates their similarities and differences from participants in the formal legal arena.

**Methods:**

The study employs a netnographic approach to analyze seven years of social media activity across 15 Facebook groups comprising over 300,000 members. Data collection included participant observation, interviews with 25 group administrators, and thematic content analysis of posts and interactions. This methodological triangulation provides a comprehensive understanding of the discourse and dynamics within these activist communities.

**Results:**

Six categories of experts were identified in the online discourse: 1. Court-admissible experts, including People directly connected to the case, people who are knowledgeable about the involved parties and the surrounding area, expert witnesses who are professionals testifying based on their field-specific expertise, and circumstantial witnesses who have experienced relevant events firsthand. 2. Non-court-admissible experts, including people with deep, self-taught expertise and people relying on nonrational sources, such as supernatural insights. The findings highlight the unique character of online activism as a dialogic space where conventional and unconventional forms of expertise coexist, contributing to public narratives around justice.

**Discussion:**

The study offers a novel conceptualization of online communities as platforms for expert-driven discourse. It underscores the importance of “pro-am” expertise and symbolic capital in shaping public understanding of contentious issues. While focused on a specific legal case, the study provides broader insights into the dynamics of expertise in online activism, emphasizing the duality of court-admissible and non-court-admissible expertise. Future research should explore these dynamics across varied contexts to further understand the role of online communities in social discourse and activism.

## 1 Introduction: social media communities as preferred arenas for activists

This article presents a novel structural conceptualization of social media communities as groups composed of various types of experts who make arguments in front of an audience. The communities that used as case studies revolve around a central legal case in Israel: the murder of Tair Rada and the trial of Roman Zadorov, who was found guilty of the murder and eventually acquitted. By and large, members of these communities think Zadorov was wrongfully convicted and call for justice for him (Lev-On, 2023b).

The focus on communities engaged in social protest in Israel is justified, among other things, because Israel has a long history of social protests based on ideology, religion, nationality, ethnicity, and other factors (Lehman-Wilzig, 1990). It is no surprise that with the enhanced use of the Internet, social media, especially Facebook, has become a central arena for mobilizing public protests, as was seen, for example, during the social protest during the summer of 2011 (Lev-On, 2020).

Facebook, the social networking site, was established in 2004 and rapidly became a central arena for information and discourse. By 2009, 2 million Israelis were Facebook members, rising to 3.4 million in 2011 and 4 million in 2013–2014 (Pereg, 2009; Elam, 2011; Kabir and Orbach, 2013). Over time, Facebook has emerged as the preferred arena for activists, especially for disadvantaged and conspiratorial groups that challenge the establishment and protest institutional injustices. These groups are often created overnight for activist purposes and help organize activity in their context (Camilli-Trujillo and Römer-Pieretti, 2017; Cammaerts, 2012; deHaven-Smith and Witt, 2013; Hara and Huang, 2011; Mehra et al., 2004). While some of these groups are barely active, others serve as a basis for continuous activism. In such cases, a community may form on the basis of social media infrastructure, consisting of people who communicate over time and share experiences, narratives, a sense of collective identity, and norms that underlie the group’s conduct (Lev-On and Hardin, 2007; Jankowski, 2006).

### 1.1 Social media activism as a community of experts

According to the literature, expertise is measured by many parameters, starting with extraordinary skills, significant and systematic knowledge in the field, ability to solve problems and face challenges and exceptional performance, history of activity and recognition by institutions and peers (Bedard and Chi, 1992; Chi, 2006; Gobet, 2016). On the Internet, expertise can be based on esoteric knowledge or symbolic status in the community or among peers.

The rise of online communities has been associated with a phenomenon some scholars term the “decline of expertise” (Nichols, 2017; Collins and Evans, 2002; Sunstein, 2006). This concept reflects a shift in which traditional experts and authorities are increasingly questioned or disregarded, with lay perspectives gaining prominence, particularly on social media platforms. In this context, expertise is no longer strictly defined by formal qualifications; instead, it can be shaped by visibility and influence within digital communities. Indeed, the findings of this study contribute to this discourse by illustrating how “professional amateurs” or “pro-ams” emerge as recognized authorities within these online communities. Note that such experts can be broadly divided into two categories: those whose expertise would be considered admissible in court and those whose authority may be recognized within online communities but would not meet formal court standards. This distinction is crucial to understanding how different types of expertise are received within formal versus informal spaces.

Leadbeater and Miller (2004) coined the term “professional amateurs” (pro-ams for short) to describe a social phenomenon that is expanding thanks to the Internet: people who are interested in a certain field, even if they do not engage in it for a living, and over time become opinion leaders about it. This phenomenon is present in many fields, from gardening and cooking to current affairs, medicine, and law (Leadbeater and Miller, 2004). These “professional amateurs” do not make a living from that field but still devote considerable time to it and publicly demonstrate proficiency and skill. The phenomenon of professional amateurs calls for a new conceptualization of the distinctions between work and hobby and between professional and amateur (see also Burgess and Green, 2018; Jenkins, 2006).

Boundary work theory focuses on the processes of creating and marking boundaries between worlds of expertise and knowledge, recognizing expertise and knowledge outside the community that produces it, and demarcating the boundary between more and less legitimate knowledge producers (Collins and Evans, 2002; Gieryn, 1999; Lamont and Molnár, 2002). The “professional amateurs” challenge the boundaries of the relevant professional communities. The current text can be seen as a mapping of strategies for challenging boundaries in online communities, focusing on the presentation of esoteric knowledge or symbolic capital.

In online communities, it is common to find people who present themselves as experts on certain topics based on interest or hobbies and who have acquired substantial knowledge over the years. Through interaction on social media, they may become opinion leaders, publishing content using professional language, presenting relevant arguments and referring to the body of knowledge accumulated in the field. They participate in dialogs, answer questions, refer to external sources of knowledge, contribute to the development of online knowledge bases, and become a focal point of symbolic capital in the community, similar to classical notions of expertise (Bedard and Chi, 1992; Chi, 2006; Gobet, 2016). However, they may lack appropriate formal training and wide recognition from colleagues and institutions.

The contribution of the present article is that it offers a novel structural conceptualization of online communities as groups consisting of different experts who make arguments before an audience. This phenomenon stood out, for example, during the spread of the COVID-19 virus, in the form of online communities that discussed health, social, political, and other issues related to the case and included the opinions of people who developed different types of skills in connection with it—sometimes based on relevant professional knowledge, sometimes less so, and sometimes even on sources of supernatural authority that gain a certain sympathy among some people (Lim, 2022; Lewenstein, 2022).

### 1.2 Discourse about justice and law in online social media

The discourse about law and justice on online social media is similar in some ways and different in other ways from the formal process in which legal issues are discussed in the courts. In the court, there are judges or juries who produce the ruling about the case. On the other hand, on social media, the conversation between the activists does not lead to a decision. In addition, the verdict given by the judges has implications on the ground in that it binds the law enforcement authorities. On the other hand, the conversation between activists may influence public opinion, but it lacks operative consequences (Grossman and Lev-On, 2023).

In addition, the formal legal process consists of two parties presenting to the judges or jurors the narrative they support and trying to convince them through findings and arguments. In contrast, the “court of social media” is characterized by one-sidedness. The arguments that are heard lean prominently toward the side that believes the formal procedure did not result in justice being done. As a result, those who read the content on social media may be exposed almost exclusively to one side, unlike what happens in the courts (Grossman and Lev-On, 2023). It is likely that this phenomenon is caused by the fact that people who are active in the context of law and justice, in most cases, are motivated by a sense of injustice and wrongdoing by the establishment. On the other hand, people who think that justice has been done are not likely to spend their time writing arguments in favor of the existing state of affairs.

Another issue that differentiates the two arenas concerns expertise. To be recognized as an expert within the legal process, one must go through a process of validation of expertise. When testimonies of people testifying by virtue of their expertise in the court of social media (for example, trackers testifying about footprints—see below) reach the court system, it treats them as experts “in their own eyes” (Case 502/07, 2010, the ruling in the first proceeding in the district court, the State of Israel vs. Roman Zadorov, p. 445). Beyond the disdain that emerges from this phrase, the intention is that this type of witness expertise has not been validated in order for it to be recognized as part of the formal procedure,

## 2 Research environment: justice for Roman Zadorov social media activism

On December 6, 2006, 13-year-old Tair Rada was found murdered at her school in Katzrin, Israel. Roman Zadorov, a flooring installer who worked at the school, was arrested 6 days later, and a week later confessed to the killing—but then immediately recanted, and has since denied any connection to the murder. Ultimately, Zadorov was convicted of murder in 2010 and sentenced to life in prison. The verdict referred to a “high-quality, dense and real fabric of evidence” that pointed to Zadorov, including his confessions to the informant and police investigators, reconstruction of the murder, and a shoe imprint on the victim’s pants that, according to the police expert, most likely originated from Zadorov’s shoe. Zadorov’s appeal to the Supreme Court was rejected in 2015.

However, the conclusiveness of the court’s ruling contradicts the court of public opinion, with polls repeatedly showing that an overwhelming majority of the public thinks that Zadorov is innocent. In 2021, a Supreme Court judge decided to grant Zadorov a retrial, and in 2023 he was acquitted (Lev-On, 2023a).

In the period immediately after the murder, the affair attracted the attention of the public, partly because the victim was a young girl murdered in the middle of the day in school. Another source that helped cast doubt on Roman Zadorov’s involvement in the murder was Tair Rada’s own mother. Shortly after Zadorov recounted how the murder was committed, she declared that she doubted whether he was indeed the killer. Over the years, problems with Zadorov’s confession and reconstruction also contributed to these doubts as well as the existence of alternative narratives about the identity of the murderer(s), the manner in which the murder was committed, and the motives behind it.

Another factor responsible for the overwhelming public interest in the case is the intensive social media activity to promote Zadorov’s innocence. Since 2009, many Facebook groups have been established that address this affair. In 2015, after Zadorov’s appeal to the Supreme Court was rejected, the number of members of these groups soared, the largest of which, “The whole truth about the murder of the late Tair Rada,” became one of the largest in Israel (Ben-Israel, 2016). The investigation materials were made available on the “Truth Today” website (from 2016). There are also a number of YouTube channels, including related video materials (including investigative videos, conversations with police informers and a reconstruction).

Apart from its scope, social media justice for Roman Zadorov activism is unique in other respects:

1. The context: This activism takes place in the context of a murder trial and a call for justice for a putative false conviction. In contrast, typically, the findings and products of police investigations and legal proceedings are far from the public eye.
2. The identity of participants in the discourse: Typically, participants in the public discourse regarding law and justice are “insiders”: police officers, lawyers, judges, reporters and legal commentators. In the Zadorov case, however, the involvement of “outsiders” is evident, including activists who are familiar with small and large issues.
3. This activism is also unique in its significant effects, for example, on public opinion of the functioning of the relevant state institutions and Zadorov’s guilt/innocence. In addition, it is unique in how it has led to the many discoveries by activists who pore through the investigation materials, including those that led to the decision to hold a retrial for Zadorov (Lev-On, 2023a).

For all these reasons, social media justice for Roman Zadorov activism represents a fascinating case for examining the characteristics and effects of this general phenomenon.

## 3 Research method

The study is based on netnographic research. Netnography is a qualitative interpretive research approach studying the behavioral and communicative patterns of people and groups online (Kozinets, 2010; Rageh and Melewar, 2013).

Netnography involves collecting data from various online sources, such as social networks, chats, and petition sites. Researchers can identify communities, observe and join them, and interview participants. The triangulation of participant observation, interviews and content analysis enables a comprehensive picture of the justice for Roman Zadorov activism. This netnographic study lasted 7 years, from December 2015 (i.e., the rejection of Zadorov’s appeal to the Supreme Court and resulting intensification of activism) until December 2022, and includes the following:

### 3.1 Observations of activism

Continuous contacts were established with group administrators and leading activists. Conversations with administrators were also about issues and dilemmas that arose regarding content that emerged in the groups and activities that took place. This was particularly helpful in learning about group splits and activist discoveries.

### 3.2 Analysis of content posted on social media

Fifteen active Facebook groups were identified, with more than 300,000 members in total. The posts in these groups and responses they elicited were documented in real time. The more active groups were sampled daily, with other groups sampled weekly. The posts that included demonstration of expertise were located and then thematically anayzed for categorizes of expertise.

### 3.3 Interviews with social media group administrators

Twenty-five interviews with admins of the various groups were conducted. The interviews included some 25 questions that addressed the general background of the interviewees, perceptions of the goals and impact of activism, questions about group management, norms, conflict resolution techniques, issues related to the kinds of people who express opinions in the groups, the kind of arguments made and how they are supported, how these arguments are treated by admins, issues related to rumors, conspiracies, smearing and bullying, and more. The interviews lasted an hour to an hour-and-a-half and were held in locations amenable to relaxed interactions such as cafes. They were conducted by four interviewers under the supervision of the lead researcher and were recorded, transcribed, and thematically analyzed.

## 4 Findings

In the findings section, I categorize six types of experts identified within the social media movement advocating for justice for Roman Zadorov. These experts fall into two main categories:

Court-admissible types of expertise: This expertise is formally recognized within judicial proceedings. The experts in this category include:

- People directly connected to the case,
- People who are knowledgeable about the involved parties and the surrounding area.
- Expert witnesses who are professionals testifying based on their field-specific expertise, and
- Circumstantial witnesses who have experienced relevant events firsthand.

Non-court-admissible types of expertise: This type of expertise, though influential in online discourse, would not meet the formal standards for court admissibility. Social media discourse includes two unique types of experts absent in formal legal settings:

- “Professional amateurs” who have developed considerable case-related expertise through independent research.
- Witnesses who do not rely on rational evidence but rather on nonrational sources, such as supernatural revelations, dreams, or similar experiences.

### 4.1 Experts on the case

Of the types of expertise reviewed in this article, the first type is “experts on their own behalf” for the case itself, as Judge Cohen, the head of the court in the original trial, called them (see above). It can be estimated that there are a few hundred activists who show a huge interest in the Zadorov case, sometimes from the beginning—and stay updated through online activity. They know the evidence. Some of them watched a significant part of Zadorov’s conversations with the informant and his investigations, including the confession and reconstruction. Following from this, the arguments they put forward are based on a long process of getting to know the materials, thinking about them, and forming an opinion.

There is a broad range of issues discussed within the groups working for justice for Zadorov, some requiring expertise in fields such as law, criminology, psychology, policing, pathology, etc. Although not formal experts in all these areas, some members have developed a deep understanding over time and can discuss various aspects related to the case using professional terminology. The groups assist in introducing activists to new areas of knowledge, arguments that can be raised, and the formal process of law and justice (Andersson and Olson, 2014; Dobos and Jenei, 2013).

**Figure 1:**
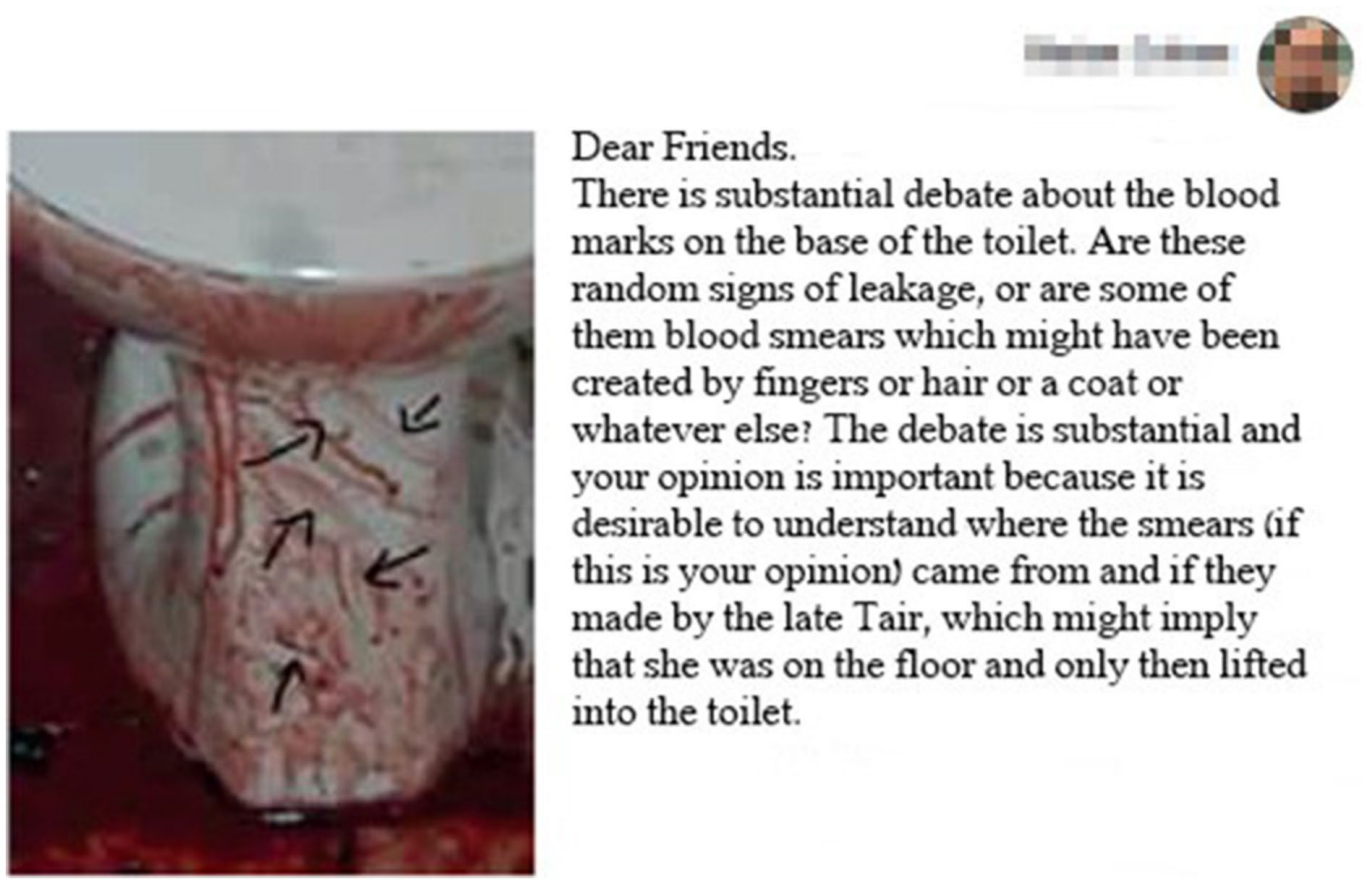
The smears of blood at the base of the toilet.

A kind of division of labor was spontaneously created among the activists (Lev-On, 2023a), according to which different activists focused on different aspects of the affair. For example, Figure 1^1^ shows the smears of blood on the base of the toilet where Tair Rada was found. The author suggests that Rada created smears on the base of the toilet “as part of desperate attempts to grab something.”

### 4.2 People who were involved in the original legal case or were connected to it in different ways

The second type of experts who participate in the social media discourse on the Zadorov case are those who were involved in the original legal case or connected to it in various ways, such as Zadorov’s family members, Rada’s friends and acquaintances, witnesses in the trial, lawyers, and many others. In the pre-social media era, people related to the case communicated primarily in courtrooms and through traditional media. However, social media has changed the rules of the game, providing a continuous and accessible meeting place for these people.

**Figure 2:**
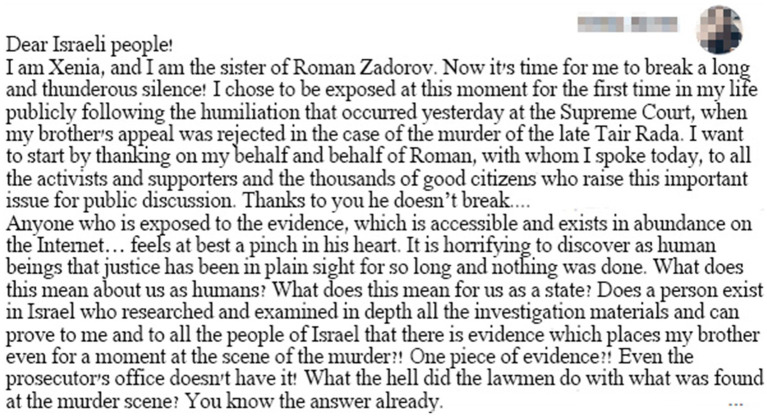
Xenia Abras breaks her silence.

An example of a person involved in the case is Olga Grishaev, the wife of Roman Zadorov. Who even served as the manager of one of the groups for a certain period, although her appointment was a “sign of appreciation” from the other group managers and Grishaev was not *de facto* involved in the management of the group. Roman Zdorov’s sister, Xenia Abras, also sometimes posts in groups on social media. After Zadorov’s appeal to the Supreme Court was rejected in 2015, she thanked the activists for supporting her brother and expressed her anger at the legal system and the police. The post was crowned one of the “posts that stirred up the web” that year (Levy, 2016) according to the MAKO website; it was shared over 4,000 times and received many comments (see Figure 2).^2^

Another example of the second type of expert involved in the case and who takes an active part in the discourse on social media is attorney Galil Spiegel, who, along with attorney David Spiegel, represented Zadorov in the first legal proceedings in the district court. In one point,^3^ she referred to the informants she requested to be called defense witnesses, whose testimony she claims was not accepted as credible. She also claims that the establishment took revenge on them and denied them benefits such as a reduction of a third of the sentence.

**Figure 3:**
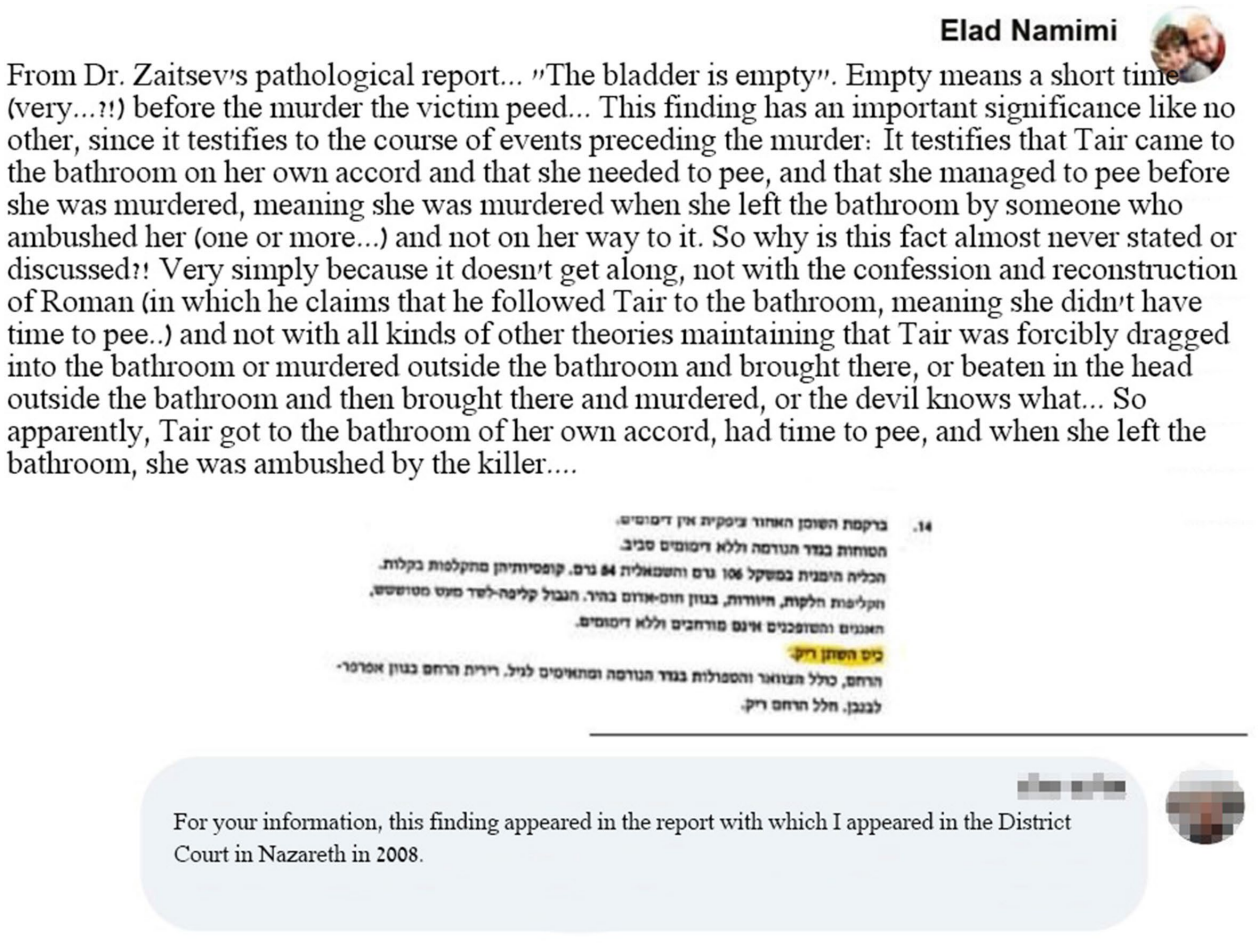
Alex Peleg argued that the bladder was found to be empty.

Another example of people involved in the original case is a response written by Alex Peleg, a former crime scene investigator who served as an expert witness for the defense. In one of his responses to the group, Peleg stated that, in his opinion, Rada’s bladder was empty at the time of the murder. He sharply criticized the court for ignoring the implications of this finding (see Figure 3).^4^

Other partners in the online discourse about the murder of Tair Rada and the Zadorov trial are Rada’s girlfriends, especially those who testified that they were in the bathroom near the time of the murder. Over the years, many on social media have accused them of being involved in the murder, even though the evidence does not point in their direction at all. Some of them shared information about the turn of events on the day of the murder and answered group member questions.

### 4.3 People who know the “central figures” or the area

The third type of experts who participate in the discourse in groups on social media are “local experts” who are not directly related to the murder case but can shed light on the “central figures” in the case, mainly Roman Zadorov and Tair Rada. In other cases, these are experts with knowledge about Katzrin, the town where the murder took place.

An example of this is a man who arguably was in prison with Roman Zadorov, who says he was arrested for 11 months, part of which he spent close to Zadorov. In one post, he testified to Zadorov’s kindness and sensitivity:

About 9 years ago I was arrested in Kishon prison and was incarcerated there for 11 months. Shortly after, Roman Zadorov arrived, who was also placed in the same cell with me. We would eat together, go to sleep at the same time together and wake up together. And that’s how I got to know this wonderful man. Really came to know him, from the inside. Because the prison is a place where you can really get to know the person… His thoughts, his fears. His hopes, and of course also his weaknesses. At his trial they said he was racist, cruel and angry. I do not know when they got to know him, because I sat next to him and during this long period he was always calm, and I never saw him get angry or quarrel with other prisoners. He would listen to everyone and help everyone, no matter what their nationality. A gentle, sensitive and kind person. This is how he also behaved to his wife on the phone, even when his situation was sad and depressing. I believe he is innocent, not because he repeated it over and over again in prison, but because that is how I knew him. A good person who is unable to hurt another. And perhaps precisely because of this kindness of his they managed to frame him.^5^

### 4.4 Expert witnesses: professionals testifying in their field of expertise

The fourth type of experts participating in the conversation on social media consists of those with relevant professional knowledge. These experts are similar to expert witnesses who are invited to give a professional opinion in their field of expertise in the legal procedure.

**Figure 4:**
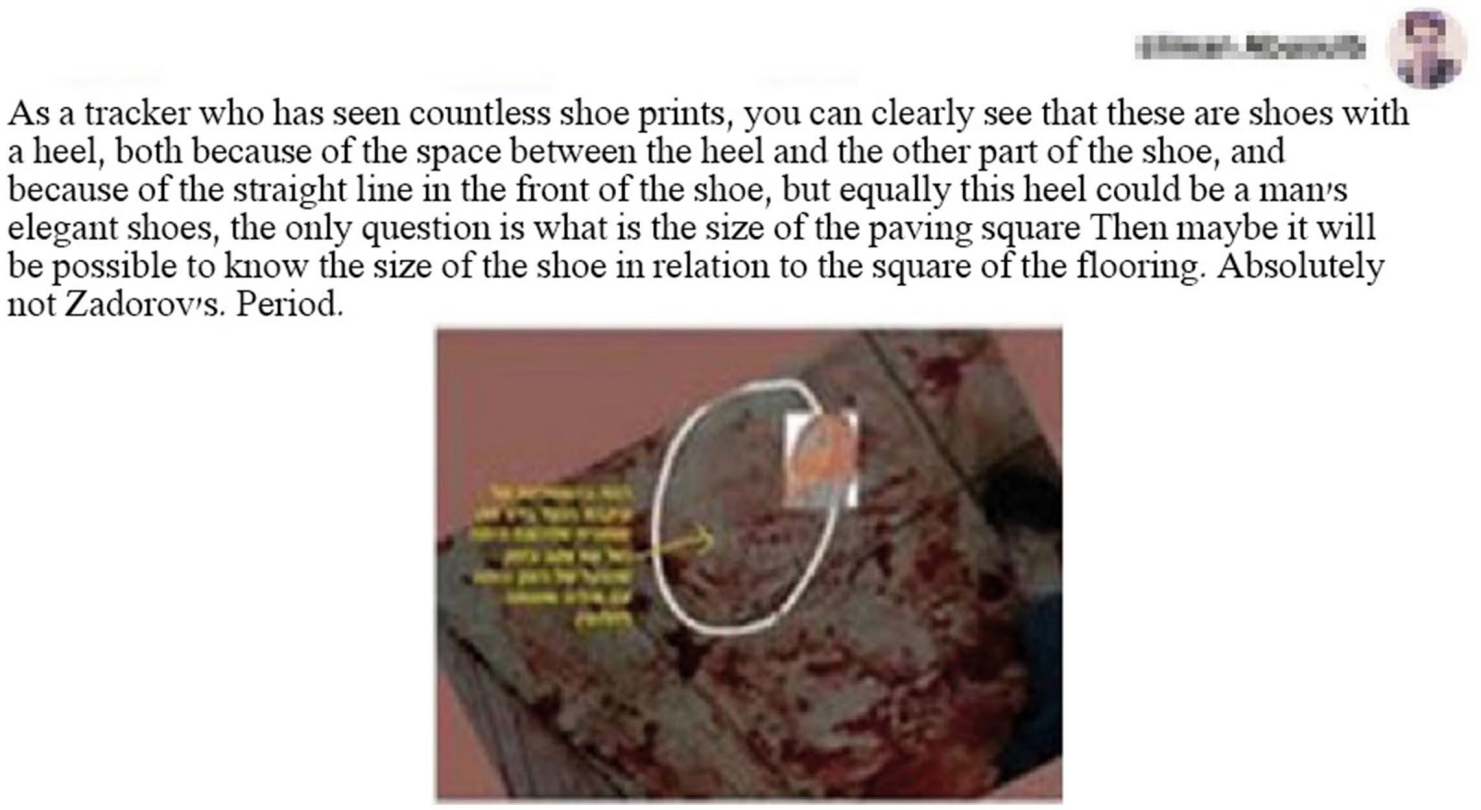
A tracker indicates the footprints of the shoes in the bathroom.

For example, a person who identifies himself as an old Bedouin tracker claimed that the shoe print in blood belongs to a shoe with a space between the heel and the back of the sole and, in particular, to a shoe whose print is different from Zadorov’s shoe print, which is shown for comparison (see Figure 4).^6^

### 4.5 Circumstantial witnesses: people who experienced events relevant to the case

**Figure 5:**
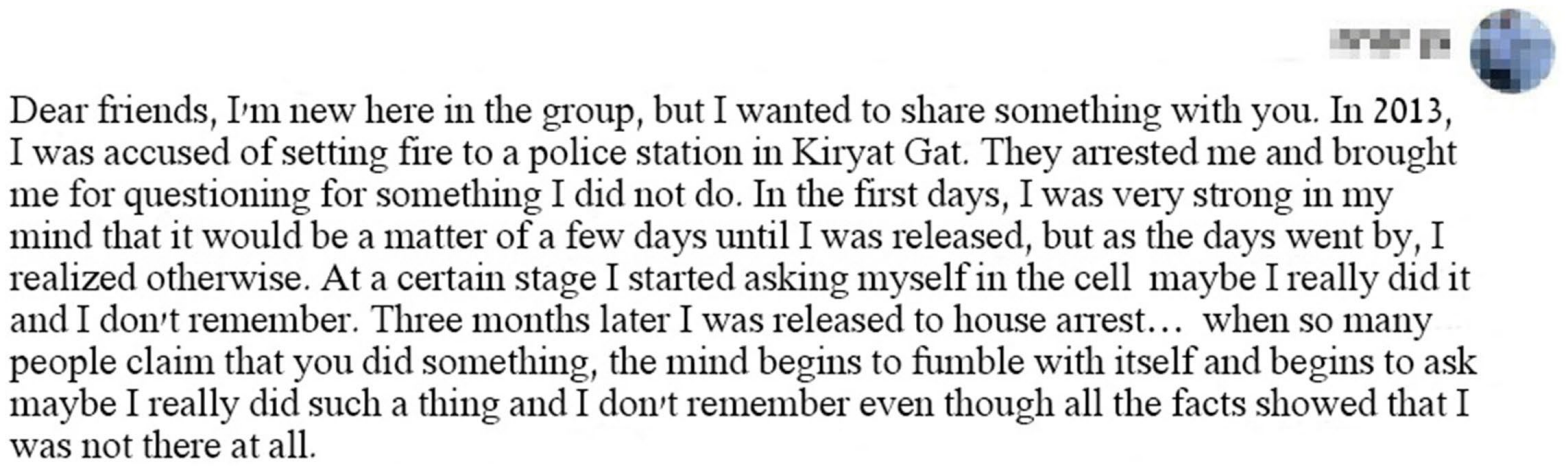
Almost confessed to a crime he did not commit.

Among the writers in the groups, one can find people who have had experiences relevant to various aspects of the case. One such testimony is that of a man (Figure 5)^7^ who was arrested in 2013 on suspicion of setting fire to a police station, which he arguably did not commit. The writer says that during the arrest, he started to “get into trouble” with himself and wonder if he might have set fire to the police station and does not remember (arguably, like what happened to Roman Zadorov). According to him, “when so many people claim that you did something, the mind begins to fumble with itself and begins to ask maybe I really did such a thing and I do not remember even though all the facts showed that I was not there at all.”

Another example is that of people who claim that the establishment has abused them (or their relatives) and that its conduct toward them is wrongful. In this category, the relatives of Amos Baranes, who was convicted of murdering the soldier Rachel Heller in 1976 and was later acquitted, stand out. In a post written by Moti Baranes, he claims that his brother fell victim to tricks similar to those use on Zadorov.^8^

### 4.6 Claims based on supernatural sources of authority

The legal arena is based on qualified sources of knowledge that are supported by evidence and pass the tests of proof and plausibility. However, the desire to uncover the truth and solve a mystery is not always fully and conclusively satisfied by this procedure, with some people turning to supernatural channels to uncover the truth. These people rely on esoteric sources of authority such as séances, fortune-telling, numerology and coffee-reading, ciphers in the Bible, and dreams.

Arguments based on these avenues also came up during the murder investigation. For example, the police collected testimonies from people who claimed to have had a séance in which dwarves reported the name “Nimrod” from Tiberias. The sender of the news “realized from the beginning” that there was no truth in it but still chose to deliver it.

**Figure 6:**
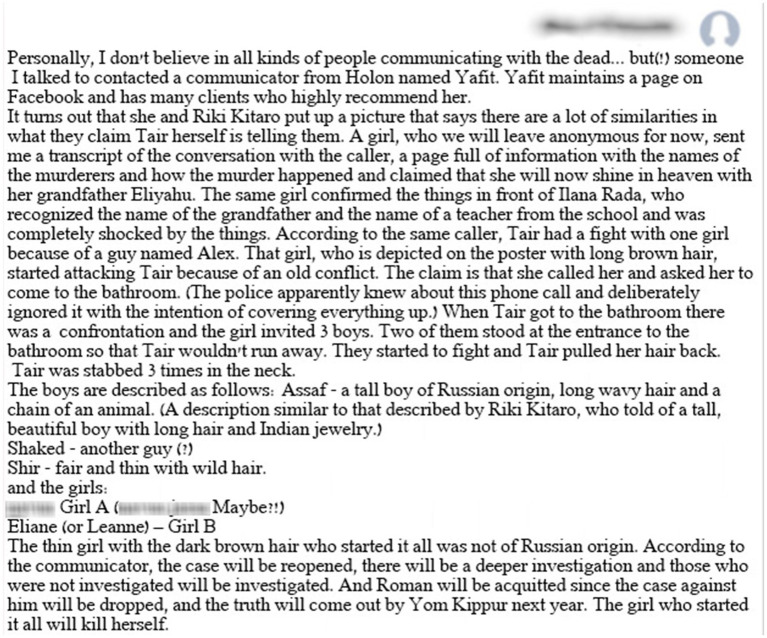
Claims based on supernatural powers.

Among the callers and dreamers, a multitude of narratives can be identified: some point to the direct involvement of teenagers in the murder, some to a drug deal to which Rada was exposed, some to the involvement of members of the Satanic cult, and more. What they all have in common is that they point to Roman Zadorov’s innocence. For example, in Figure 6,^9^ there is a conversation with a spirit medium to whom Rada’s ghost reportedly recounted in great detail the identity of those involved in the murder and how it was carried out.

## 5 Discussion and conclusions

This article offers a novel structural conceptualization of communities on the Internet as groups consisting of various experts who argue before an audience, relying on the case study of the discourse in social media groups calling for justice for Roman Zadorov. By examining the conversations, speakers, and expertise attributed to them in the groups, it is possible to propose a useful typology of several categories of experts who participate in this kind of discourse. Such “pro-am” experts perform “boundary work” (Collins and Evans, 2002; Lamont and Molnár, 2002; Gieryn, 1999) due to the esoteric knowledge they created or the symbolic capital they accumulated.

Over the years, online social media has become the preferred arena for activists in general, specifically for disadvantaged groups and groups with a conspiratorial “aroma” that challenge the establishment and protest institutional injustices. Such groups are created overnight for activist purposes and help organize related expressions of activism. Sometimes these are groups or pages that are set up *ad hoc* and have little activity. In other cases, they are the basis for ongoing activism among a group of people interested in a cause. In such cases, a community may form using social media infrastructure—a group of people who communicate over time, and, based on their interaction, a collection of shared experiences, narratives regarding joint action, a sense of collective identity, and norms is formed.

This article illustrates the importance of expertise in these processes. Expertise is measured in many contexts, starting with extraordinary skills, significant and systematic knowledge in the field, the ability to solve problems and face challenges, exceptional performance, history of activism, and recognition by institutions and peers (Bedard and Chi, 1992; Chi, 2006; Gobet, 2016).

One of the prominent findings that emerged from the ethnographic research concerns the presence of people who function as experts who specialize in various central themes address by the community and present their arguments to the social media audience. Thus, the groups on social media dealing with the Zadorov affair serve as gathering points for people with unique relevant knowledge: people connected to the affair, people who know the persons involved and the area, professionals testifying in their field of expertise, and circumstantial witnesses who experienced relevant events. In addition, the discourse on social media includes two types of experts without a counterpart in the formal procedure: people who have gained expertise regarding the case and witnesses who do not rely on rational arguments but on non-rational esoteric knowledge and dreams.

The significant discourse that develops based on the words of the various experts sheds light on the community as a dialogic space where a significant part of the discourse occurs through the interaction between experts of different types and the audience. The distinction between court-admissible and non-court-admissible expertise highlights the unique dynamics of online communities, where both conventional and unconventional sources of authority coexist. This arrangement suggests that digital spaces enable the public to form opinions outside formal legal parameters, potentially influencing societal views on justice through a broader and sometimes non-evidentiary lens.

A critical limitation of this study lies in its scope, which is confined to online communities specifically engaging with perceived injustices. This is a unique type of online community where both lay and professional expertise can gain traction and authority. In contrast, not all online communities exhibit the same dynamics, particularly those that do not address high-stakes, contentious issues. Consequently, while this study sheds light on the mechanisms by which ‘pro-am’ expertise shapes collective discourse, its applicability may be limited to contexts where the community’s central issue has sufficient public resonance to attract various stakeholders, including professionals and quasi-experts. Follow-up studies can further examine this issue through the analysis of content in different social network groups operating in different contexts, as well as through interviews with the experts and the audience in these groups, to understand their perception of social network groups as places for dialog between experts and audiences.

## Data Availability

The raw data supporting the conclusions of this article will be made available by the authors, without undue reservation.

## Footnotes

^1^We demand a fair trial in the case of the murder of the late Tair Rada, 7/28/2017.

^2^The whole truth about the murder case of the late Tair Rada, 24.12.2015.

^3^The whole truth about the murder case of the late Tair Rada, 2016.

^4^The truth today - in memory of the late Tair Rada, 26.6.2016.

^5^We demand a fair trial in the case of the murder of the late Tair Rada, 2.12.2015. In a conversation with Roman Zadorov (November 2021), he verified that this is indeed a person who was with him in prison for a certain period of time.

^6^The whole truth about the murder case of the late Tair Rada, 12/30/2015.

^7^The whole truth about the murder case of the late Tair Rada, 18.4.2017.

^8^The whole truth about the murder case of the late Tair Rada, 18.1.2016.

^9^The mystery of the double murder in Katsrin, 2016.
